# Local Community Response to Mass Asymptomatic COVID-19 Testing in Liverpool, England: Social Media Analysis

**DOI:** 10.2196/34422

**Published:** 2022-08-04

**Authors:** Charlotte Robin, Charles Symons, Holly Carter

**Affiliations:** 1 Behavioural Science and Insights Unit UK Health Security Agency Liverpool United Kingdom; 2 Behavioural Science and Insights Unit UK Health Security Agency Bristol United Kingdom; 3 Behavioural Science and Insights Unit UK Health Security Agency Salisbury United Kingdom

**Keywords:** COVID-19, asymptomatic testing, social media, attitude, behavioral science, testing, behavior, community, England, acceptance, barrier, motivator, hesitancy, communication

## Abstract

**Background:**

Mass asymptomatic testing for COVID-19 was piloted for the first time in the United Kingdom in Liverpool in November 2020. There is limited evidence on uptake of mass testing, and previously where surge testing has been deployed, uptake has been low.

**Objective:**

There was an urgent need to rapidly evaluate acceptance of asymptomatic testing, specifically identifying barriers and facilitators to taking part.

**Methods:**

As part of the wider evaluation, we conducted a rapid thematic analysis of local community narratives on social media to provide insights from people unlikely to engage in testing or other standard evaluation techniques, such as surveys or interviews. We identified 3 publicly available data sources: the comments section of a local online newspaper, the city council Facebook page, and Twitter. Data were collected between November 2, 2020, and November 8, 2020, to cover the period between announcement of mass testing in Liverpool and the first week of testing. Overall, 1096 comments were sampled: 219 newspaper comments, 472 Facebook comments, and 405 tweets. Data were analyzed using an inductive thematic approach.

**Results:**

Key barriers were accessibility, including site access and concerns over queuing. Queues were also highlighted as a concern due to risk of transmission. Consequences of testing, including an increase in cases leading to further restrictions and financial impact of the requirement for self-isolation, were also identified as barriers. In addition, a lack of trust in authorities and the test (including test accuracy and purpose of testing) was identified. Comments coded as indicative of lack of trust were coded in some cases as indicative of strong collective identity with the city of Liverpool and marginalization due to feeling like test subjects. However, other comments coded as identification with Liverpool were coded as indicative of motivation to engage in testing and encourage others to do so; for this group, being part of a pilot was seen as a positive experience and an opportunity to demonstrate the city could successfully manage the virus.

**Conclusions:**

Our analysis highlights the importance of promoting honest and open communication to encourage and harness existing community identities to enhance the legitimacy of asymptomatic testing as a policy. In addition, adequate and accessible financial support needs to be in place prior to the implementation of community asymptomatic testing to mitigate any concerns surrounding financial hardship. Rapid thematic analysis of social media is a pragmatic method to gather insights from communities around acceptability of public health interventions, such as mass testing or vaccination uptake.

## Introduction

As part of the United Kingdom’s response to COVID-19, in September 2020, the government announced a large-scale expansion of the national testing program, with the intention of regular testing of the entire UK population on a weekly basis, regardless of symptoms [1]. This strategy was known as “Operation Moonshot” and involved using lateral flow antigen tests, which aim to provide results within 30 minutes.

To pilot the operationalization and effectiveness of mass testing, on November 2, 2020, it was announced that Liverpool City would be offered asymptomatic testing for everyone who lived or worked in the city, before the rest of the country. The pilot was a collaboration between the National Health Service (NHS) Test & Trace, Liverpool City Council (LCC), NHS Liverpool Clinical Commissioning Group, the Army (8 Engineer Brigade), Cheshire & Merseyside Health & Care Partnership, and Liverpool Charity and Voluntary Services.

A similar mass asymptomatic testing pilot in Slovakia resulted in extremely high uptake of testing, with 97% of eligible people taking part in the mass testing pilot, resulting in 38,000 new cases being identified within 2 days [2]. In the United Kingdom, research suggests that, although intentions to take a test if asymptomatic were quite high at the time [3], actual engagement with mass asymptomatic testing during the Liverpool pilot was much lower (25%) [4]. There was an urgent need to rapidly gather local insights in the Liverpool city community, to understand barriers and facilitators to engaging with mass asymptomatic testing and to inform ongoing engagement and communication strategies to increase uptake of mass asymptomatic testing. To address this and to supplement self-report survey data, we carried out rapid thematic analysis of local narratives from local community media and social media sites in Liverpool. This work was part of a wider evaluation of the Liverpool mass testing pilot, known as MAST (mass, asymptomatic, serial testing), that was led by The University of Liverpool with NHS Test and Trace, Public Health England (PHE), the Joint Biosecurity Centre, and Office for National Statistics [4]. The pilot resulted in 25% of residents taking part in testing using lateral flow tests and the identification of 897 COVID-19 cases. The aim of this study was to identify barriers and facilitators to engaging in mass asymptomatic testing and to generate recommendations for improving uptake of mass asymptomatic testing in future.

## Methods

### Aim

As part of the wider evaluation work undertaken by the Evaluation Steering Group, we conducted a rapid thematic analysis of local narratives from local community media and social media sites in Liverpool. The aims of this analysis were to provide insights into local narratives surrounding MAST, particularly from people who may not engage in testing or other standard evaluation techniques such as surveys and interviews; to inform a broader understanding of public test-seeking behaviors, including facilitators and barriers to accessing testing; and to optimize management of mass testing as part of the national COVID-19 response.

### Population

Liverpool is a city in the northwest of England, with a population of around 500,000. The average age of the population is 37.6 years [5], and in 2019, it was ranked the third most deprived local authority area in England based on the overall Indices of Multiple Deprivation score [6]. In week 41 2020 (October 5 to October 11), just before the end of a national lockdown and prior to the implementation of a new tiering system for COVID-19 restrictions, Liverpool had one of the highest rates of COVID-19 in England (659 per 100,000) [7] and was the first area of England to be placed under very high alert (Tier 3) restrictions on October 14, 2020. A strong sense of identity and belonging exists in Liverpool communities, reinforced by experiences with racism, stigma, and marginalization [8,9].

### Ethics Approval

In line with British Psychological Society guidelines [10] for conducting internet-mediated research, this research did not require ethical approval because only publicly available data (comments posted in response to public Facebook posts, Twitter posts, or comments posted in relation to online media articles) were used. The PHE Research Ethics and Governance Group was consulted and confirmed that ethical approval was not required for this research.

### Sampling

Data were collected from publicly accessible sources of community narratives, including social and online media sites. These included online comments sections from the local online newspaper for Liverpool City, which has a large circulation, the LCC Facebook page, and Twitter. Sampling captured comments posted from November 2, 2020 (when Liverpool was announced as the city to pilot mass testing) to November 8, 2020, to cover the period before and during the first week of the pilot. All publicly accessible comments on identified posts or articles were copied and pasted to text documents for coding.

Articles from the local online newspaper about the mass testing pilot were identified using the search terms “testing” and “mass testing” between November 2, 2020, and November 8, 2020. The searches resulted in the identification of 11 articles, and all comments from these articles were sampled for analysis.

All posts made by the council on the LCC Facebook page related to the mass testing pilot were identified between November 2, 2020, and November 8, 2020. Overall, 16 posts were identified, and all comments from other Facebook users on these posts were sampled for analysis.

The following search string was used to search Twitter to identify tweets with the hashtags #liverpooltesting and/or #masstesting between November 2, 2020, and November 8, 2020, sent in the Liverpool area: *near:liverpool (#liverpooltesting OR #masstesting) until:2020-11-08 since:2020-11-02*.

The search included replies to tweets (which may not necessarily have originated in or near Liverpool) and tweets containing links. In addition to the hashtag search outlined in the previous paragraph, all replies to 2 tweets announcing the mass testing pilot (1 from the local newspaper and 1 from LCC) before 00:00 on November 8, 2020, were collected. Replies to tweets from official accounts (eg, LCC and news media sources) were included in Twitter data collection, but the original tweets were excluded from analysis as they reflected official, organizational perspectives, rather than lay, public perspectives.

Overall, 1096 comments were sampled: 219 newspaper comments, 472 Facebook comments, and 405 tweets.

### Analysis

Data were depersonalized by removing any identifiable data (including names and locations) and divided and analyzed separately by 2 authors in NVivo or Microsoft Word. An inductive approach using open coding [11] identified key themes of interest and was used to develop the initial coding framework. During this stage, meetings were held to discuss coding and reach consensus. Through this process, the authors developed a final coding framework using the framework approach, a type of thematic analysis that is commonly used in research that has implications for policy [12]. This coding framework was then applied to the remaining data. Data were categorized into 2 broad themes of interest (facilitators to testing and barriers to testing), each of which was then divided into relevant subthemes.

## Results

### Facilitators to Getting Tested

For those motivated to get tested, key drivers were a desire to protect the community, a belief that mass testing could help the city return to normality, and a belief that testing would be (or experience that testing was) convenient and efficient. Some example quotes are included in the following sections, and further examples are shown in Table 1.

**Table 1:** Facilitator and barriers to engagement with mass asymptomatic testing in Liverpool (2020)—results of the thematic analysis of social media.

Theme	Example quote	Source	Date
**Facilitator: protecting the community**			
	“No symptoms just want to do my bit!”	Facebook	November 7, 2020
	“Get in and get it done. Save a life maybe?”	Online newspaper	November 6, 2020
	“I actually got tested so I can be sure I’m not a carrier infecting others! It’s a bit insulting to assume every person from Liverpool is just getting tested so they don’t have to work.”	Facebook	November 7, 2020
	“If it saves lives and gets this city back to some semblance of normality then I am all for it.”	Online newspaper	November 3, 2020
	“I think it’s perfectly reasonable to be careful in Liverpool when 1 in 250 people currently have the virus (many of whom will not know about it) and the risks for vulnerable people are much greater. Why wouldn’t you follow the advice from people who’ve dedicated careers to this?”	Twitter	November 3, 2020
	“I will be taking part in #MassTesting #Liverpool to break the chain of transmission and protect the people I love.”	Twitter	November 7, 2020
	“Protecting or vulnerable is most important. With regular testing, we can get tested on a Friday after work, if negative go see, spend time with vulnerable loved ones, and then return to work and school etc, repeat until necessary.”	Facebook	November 5, 2020
**Facilitator: return to normality**			
	“It's important we get this testing and tracing working effectively so that we can go back to ‘normal’ life. People need to recognise their own responsibility though and self-isolate when appropriate.”	Facebook	November 6, 2020
	“The more tests, the more people that will be diagnosed, the quicker we can put a cap on it in LIVERPOOL, the more likely WE (not London, not the Tories) but WE, will get out before [Christmas].”	Online newspaper	November 3, 2020
	“If everyone gets tested 2 or 3 times a week we'd potentially have very very few cases in a matter of weeks.”	Online newspaper	November 5, 2020
	“Just get yourself tested, and then we can all start to think about getting back to normal. You can’t be anti-lockdown and anti-testing.”	Twitter	November 2, 2020
**Facilitator: positive experience**			
	“Hand it in, results back in 40 mins #covid #Liverpooltesting”	Twitter	November 6, 2020
	“Arrived at the test centre, got tested, and received results through all within one hour. Well done to all the Army and NHS staff involved”	Twitter	November 7, 2020
	“...once I had the test it took under an hour for the result to come through ...so this test could be a game changer ...test wait and if negative fly or entry to a theatre etc”	Facebook	November 6, 2020
**Facilitator:** **shared social identity with others in Liverpool and with authorities**			
	“Let's support them. What I have always loved about Liverpool is the community spirit, warmth and the way we pull together in a crisis…If it works it will have positive effects not just in Liverpool but across the whole country. All eyes are on us. Let's show the country that Liverpool can beat covid 19, and they can too.”	Online newspaper	November 3, 2020
	“Really great to see such a huge positive response to this - together we will do this. Well done Liverpool [thumbs up emoji].”	Facebook	November 6, 2020
	“Absolutely, come on Liverpool we have got this!”	Twitter	November 4, 2020
	“We are the test bunnies, but this isn’t a negative thing, in fact, if we get this sorted, we’ll be the first back to normal.”	Online newspaper	November 3, 2020
**Barriers: practical barriers**			
	“I wouldn't bother booking, having a time slot doesn't make any difference, you have to queue up with everyone else, it’s a joke. [In response] Might not bother at all then if it’s not organized.”	Facebook	November 6, 2020
	“3 1/2 hour wait at *[location]* even though I booked! Didn’t bother waiting, won’t bother again! [angry face emoji].”	Facebook	November 6, 2020
	“I am in a family of 6, can only order 4 home tests. Why?”	Facebook	November 6, 2020
	“Tried to book a test, via http://gov.uk link but only allowing me to go for a test in [*location*]? As a key worker in Liverpool this doesn’t make sense... not sure this system is ready to be rolled out yet...”	Twitter	November 6, 2020
	“…they will never help us all. I won't be getting tested unless I'm unwell as I don't get paid for being off and not entitled to those payments. I am sure lots won't. Hopefully mass testing certain groups will. Pin point the problem anyway [thumbs up emoji]”	Facebook	November 4, 2020
	“…Not just the self employed. I don’t earn enough to qualify for sick pay but my wages are needed to keep us afloat. And because we don’t claim benefits, we can’t get that payment from the council. I’ve already had to isolate through track and trace and lost 2 weeks wages. Like you, no help available anywhere. I want to participate because I believe testing is the only way out of this hole but, yet again, it’s the people who actually work who lose out.”	Facebook	November 4, 2020
	“Well I went wrong one. Still had test but the normal one. I have to make sure I do the right one next time [eye roll, laughing face emoji].”	Facebook	November 6, 2020
	“Queues for booked appointments at [*location*] are hours long. There is no signage there are people leaving the queue having waited over 2 hours.”	Facebook	November 6, 2020
**Barrier: risk of transmission**			
	“How do we know these test centers aren't spreading 'it'.”	Facebook	November 2, 2020
	“I just wouldn't do that wait in a queue like that it's pathetic and more to the point riskier”	Facebook	November 6, 2020
	“It infuriates me that the PM positively encouraged these awful tests where you are more likely to get pneumonia stood in a queue, in the cold, without a mask.”	Facebook	November 6, 2020
	“Being at the testing site today it would seem that there are two 'sites' in the same car park (one for invited people with symptoms and one for asymptomatic people who booked a test) and I was in a single queue of both groups mixed because of no direction from staff or signs”	Twitter	November 6, 2020
	“What a shambles! Wouldn't let me book a drive in test so I booked a walk in test for 1pm. The que [sic] is absolutely huge, nobody knows what's going on. No managers just car park assistants to ask. Why give so many people the same time? No social distancing. I've walked away” “More chance of me getting covid with that system. Please look at the numbers you are allowing to book in the half hour slots #farcical”	Twitter	November 6, 2020
**Barrier: perceived ineffectiveness of testing**			
	“Don’t see the point you could get tested today and all clear and then catch it tomorrow.”	Online newspaper	November 6, 2020
	“How does a Covid test help you get better from the virus? We are in Lockdown so we are all isolating anyway.”	Online newspaper	November 6, 2020
	“What’s the point? They can’t cure it!!!”	Online newspaper	November 3, 2020
**Barrier: lack of trust**			
	“The tests are not accurate & not fit for purpose, giving up to 85% false positives, they do not isolate Covid, so what's the point in getting tested, just doesn't make sense to me?”	Online newspaper	November 6, 2020
	“I won’t be getting tested or using the so-called NHS app whilst Serco are involved.”	Twitter	November 2, 2020
	“Forced tests today, forced vaccines tomorrow.”	Twitter	November 2, 2020
	“Not a chance in hell would I get one of these tests.... corrupt government!”	Twitter	November 2, 2020
	“Precisely that...every dog on the street know the tests are wholly unreliable, and the possibility of false negatives high, and yet everything that even meekly questions the narrative is a conspiracy theory!! Dismal!!”	Twitter	November 3, 2020
	“I wonder if that will increase the so-called number of cases They can use those false cases to justify their lockdown #WakeUp”	Twitter	November 2, 2020
	“Liverpool are being played for mugs. The disease rate is falling by itself. They are going to find a lot of “cases” to justify the lockdown.”	Twitter	November 2, 2020
	“More tests = More False Positives More false positives = More False ‘cases’ More cases = More lockdown restrictions More lockdowns = More power to the government More government power = Less rights & less liberty for the UK people STOP GETTING TESTED”	Twitter	November 3, 2020
	“Don’t get tested. Dodgy test = false positives = further lockdown”	Twitter	November 6, 2020
	“The will make the R rate rise and we won't get out of lockdown”	Facebook	November 5, 2020
	“Imagine how many old criminal cases that will be solved with the mass DNA harvest.”	Twitter	November 3, 2020
	“Can Liverpool City Council explain why they are taking peoples DNA. That's what the test is isn't it?”	Facebook	November 7, 2020
**Barrier: shared social identity with others in Liverpool but not with authorities**			
	“Surprise Surprise, we are just one big test case.”	Online newspaper	November 7, 2020
	“Mass testing should be in London, not Liverpool. The Greater Manchester mayor said no to our area being treated like a canary in a coal mine.”	Facebook	November 6, 2020
	“The guinea pigs are staying in their cages”	Twitter	November 6, 2020
	“Why not do this in London first.”	Twitter	November 2, 2020
	“We are the Guinea Pigs for everything”	Twitter	November 2, 2020
	“Operation Scouse Guinea pigs is a go.”	Twitter	November 2, 2020
	“Do you really think they want Liverpool out of tier 3?? If they wanted anyone out of tier 3 Liverpool would be bottom of the pile.”	Facebook	November 6, 2020
	“There's an agenda behind this and obviously us being the guinea pigs isn't a coincidence. That toffee nosed slob hates Liverpool and would love to bring the city to its knees. If he'd moved his backside in before March and locked down sooner, maybe we wouldn't be at this point- unless this was all by design, I don't know anymore.”	Online newspaper	November 3, 2020
	“There [sic] blaggin ya eds [your heads] big time, look if you do what we say we can save [Christmas] for you!!!”	Online newspaper	November 2, 2020
	“Don't comply, we're being scapegoated again, you will gain nothing by being tested apart from losing your jobs and having your kids barred from school, it's just another money spinner for the old school tie network of the Eton set”	Online newspaper	November 3, 2020
	“'Obedient biodrones’ couldn't agree more.“	Online newspaper	November 6, 2020
	“People who don't question what's going on just play into the hands of the greedy politicians and snearing [sic] middle classes. The problem is the rest of us are left to protest and fight for their rights as well. Come on people, please wake up.”	Online newspaper	November 5, 2020
	“Won’t be testing me anytime soon, I’m no government clone, bring them swabs anywhere near me and they’ll be inserted where the sun don’t shine”	Twitter	November 2, 2020
	“The car park was full of SERCO workers who told me they had been drafted from London and the South and knew nothing about local details. Couldn’t you get local Test & Trace workers?”	Facebook	November 6, 2020

#### Protecting the Community

Wanting to protect their community was a key motivator for those who engaged with the testing program. This included a motivation to protect their loved ones, which in turn would have wider implications for public health:

I will be taking part in #MassTesting #Liverpool to break the chain of transmission and protect the people I love.

Twitter, November 7, 2020

There was also a wider understanding of community, beyond immediate family and friends. For example, people wanted to protect vulnerable people, both within their family and elsewhere. Within this, they fulfilled a sense of duty and felt, by engaging with the asymptomatic testing, they were contributing to saving lives:

Get in and get it done. Save a life maybe?

Online newspaper, November 6, 2020

#### Return to Normality

Tied in with wanting to protect the community was the anticipation of being able to return to “normal”:

If it saves lives and gets this city back to some semblance of normality then I am all for it.

Online newspaper, November 3, 2020

There was an understanding that pulling together as a community would not only help protect others and save lives but would also help the city recover quicker, specifically reducing the number of cases and entering a lower tier following the national lockdown:

The more tests, the more people that will be diagnosed, the quicker we can put a cap on it in LIVERPOOL, the more likely WE (not London, not the Tories) but WE, will get out before [Christmas].

Online newspaper, November 3, 2020

#### Positive Experience

Among commenters who did get tested, some discussed positive experiences of the testing process itself. These positive experiences were noted throughout the testing process, including ordering tests or booking test slots. Positive experiences were also shared for the time spent at the test site, specifically how organized the process was:

Arrived at the test centre, got tested, and received results through all within one hour. Well done to all the Army and NHS staff involved.

Twitter, November 7, 2020

In addition, the kindness of the staff working at the test centers was noted as part of their positive experience of the end-to-end test experience.

#### Shared Social Identity With Others in Liverpool and With Authorities

There is a strong sense of social identity associated with the city of Liverpool; the city is who people are and where they belong. Where people identified with others in the city, as well as with authorities managing the response, shared identity operated not only as an individual motivator to get tested but also to encourage others to do the same:

Absolutely, come on Liverpool we have got this!

Twitter, November 4, 2020

There was a sense of wanting to come together as a community, to help not only the city but also the rest of the country. Rather than seeing this as a sacrifice on behalf of the rest of the country, it was seen as an opportunity to demonstrate that Liverpool can successfully manage the virus, setting an example for everyone else:

Let's support them. What I have always loved about Liverpool is the community spirit, warmth and the way we pull together in a crisis…If it works it will have positive effects not just in Liverpool but across the whole country. All eyes are on us. Let's show the country that Liverpool can beat covid 19, and they can too.

Online newspaper, November 3, 2020

Rather than being chosen as the city to pilot test being viewed as negative, the feeling of social identity and an emotional connection with the city helped people understand the pilot as an opportunity and privilege for the city, for example being the first place out of lockdown or into a lower tier following the end of the national lockdown.

### Barriers to Getting Tested

Analysis of the data highlighted several barriers to people getting tested. The key barriers identified were practical barriers to testing, concern over the risk of transmission at the testing sites, and lack of trust in the mass testing program and in the government.

#### Practical Barriers

A key practical barrier to getting tested was inconvenience associated with attending testing sites. Various factors associated with inconvenience were identified, including long queues at testing sites and poor organization of the testing process:

3 1/2 hour wait at [location] even though I booked! Didn’t bother waiting, won’t bother again! [angry face emoji].

Facebook, November 6, 2020

In addition, there was frustration that the booking system did not help to reduce queue length on attending the testing site—those who experienced long queues despite advanced booking were less motivated to try again. In some cases, people shared their negative experiences on social media, for example around queues, disorganization, or delays in getting results; this may have influenced others’ decisions in regard to getting a test.

The uncertainty surrounding the pilot, particularly in the first few days of launch, led to questions being raised in local narratives. These were predominantly related to access to testing, how to book, where the test sites were, whether there were separate sites for asymptomatic testing, and who would be conducting the tests. Uncertainty around how to access testing sometimes resulted in people attending the wrong test centers and having the wrong test or being unable to book tests at all:

Well I went wrong one. Still had test but the normal one. I have to make sure I do the right one next time [eye roll, laughing face emoji].

Facebook, November 6, 2020

Another practical barrier to getting tested related to concerns about the consequences of someone testing positive. For example, some individuals raised lack of compensation if required to self-isolate following a positive test as a reason for not getting tested.

#### Risk of Transmission

As well as long queues being a barrier to accessing testing because of the inconvenience, they also contributed to concerns over the risk of transmission. For some, the risk of catching COVID-19 while queuing was cited as a reason for not wanting to get tested:

I just wouldn't do that wait in a queue like that it's pathetic and more to the point riskier.

Facebook, November 6, 2020

In some cases, commenters who had participated in testing reported lack of distancing at test sites, with symptomatic people having to queue alongside asymptomatic people.

#### Perceived Ineffectiveness of Testing

There was also confusion surrounding the purpose of mass testing and how it would help the overall COVID-19 response. In addition, there was the perception that there was no practical purpose for getting tested because there would be no individual benefit to knowing your disease status, particularly if asymptomatic:

What’s the point? They can’t cure it!!!

Online newspaper, November 3, 2020

#### Lack of Trust

In addition to the more passive barriers outlined in the previous sections, there was a motivation to actively avoid participation in mass testing, sometimes expressed alongside discouragement to others or criticism of fellow residents who had been or were planning to get tested. A key factor motivating people to not get tested was lack of trust. This included lack of trust in the accuracy of the test and lack of trust in stakeholders involved in the delivery of mass testing, such as national and local government, scientists, and Test and Trace:

Not a chance in hell would I get one of these tests.... corrupt government!

Twitter, November 2, 2020

Those who displayed low trust in the mass testing process, and in government response generally, raised potential illegitimate bases on which testing was implemented or highlighted potential adverse consequences of mass testing for Liverpool. These potential consequences focused on 2 main concerns: coercion by the state during mass testing and further restrictions following mass testing due to the rise in the number of known cases. The latter concern was related to the aforementioned lack of trust in the accuracy of the test, with commentators predicting an anticipated high number of false positive cases (sometimes referred to as a “casedemic”) that would lead to further restrictions in Liverpool only, including a prolonged lockdown:

More tests = More False Positives. More false positives = More False “cases”. More cases = More lockdown restrictions. More lockdowns = More power to the government.

Twitter, November 3, 2020

Other drivers for not getting tested were concern about the use of mass testing for surveillance or DNA gathering:

Can Liverpool City Council explain why they are taking peoples DNA. That's what the test is isn't it?”

Facebook, November 7, 2020

#### Shared Social Identity With Others in Liverpool But Not With Authorities

Analysis highlighted how social identity can have a dual role in understanding responses to testing. For those who identified with authorities managing the response, as well as with others in the city, this operated as a facilitator to getting tested (as described in the previous sections). However, for those who did not trust the government response and for whom there was no shared identity with authorities, shared identity with others in the city contributed to motivations not to get tested. In this instance, people felt that mass testing was something being imposed on them rather than something they could engage with as a community:

Surprise Surprise, we are just one big test case.

Online newspaper, November 7, 2020

This led to a sense of marginalization; local communities felt disconnected from those making the decisions, particularly central government. Feeling disenfranchised from local and central government resulted in discussions around ulterior motives, highlighting a breakdown in trust between the local community in Liverpool and those in power:

Do you really think they want Liverpool out of tier 3?? If they wanted anyone out of tier 3 Liverpool would be bottom of the pile.

Facebook, November 6, 2020

In addition, the role of social identity in local narratives around testing resulted in some members of the community not wanting to conform with what others were doing. For this group, people who were participating in testing were viewed negatively; they had lost their identity and become “other” and therefore outsiders in the local community, which resulted in criticism for “conforming”:

‘Obedient biodrones’ couldn't agree more.

Online newspaper, November 6, 2020

Social identity also played a part in concern over “outsiders” coming to the city to deliver the testing program and highlighted a lack of trust in central government.

## Discussion

### Principal Findings

Findings from this study have implications for the management of mass testing in the future, both in terms of practical management of setting up and running testing sites and communication with members of communities in which mass testing will be provided. Our analysis identified that facilitators for engaging in mass asymptomatic testing included a sense of community, a desire to return to normality, positive experiences of others, and having a shared identity with Liverpool authorities. Barriers included practical barriers (access to test sites, long queues), concern over risk of transmission, perceived ineffectiveness of testing, lack of trust, and a shared social identity with the Liverpool community but not those in authority.

Findings showed that one of the key motivators to engaging with the pilot in Liverpool was a strong sense of community identity and belonging, both with city residents and local authorities. However, when a strong sense of identity was not shared with authorities (for example, where local and central governments were not seen as trusted organizations), community identity acted as a barrier to engagement with testing. Furthermore, it actively motivated people to disengage from the pilot. To ensure that shared community identity acts as a facilitator rather than a barrier, it is important that members of the community identify with the authorities managing the testing, as well as identifying with each other. This is in line with previous research that emphasized that shared identity is a crucial part of promoting community resilience in response to mass disasters and emergencies [13] and can provide a basis for understanding of the relationship between communities and authorities [14]. Harnessing and working with existing shared identities, such as the identity shared by Liverpool city residents, can help build and maintain trust in authorities and the information they provide [14]. Authorities should communicate openly and honestly and demonstrate respect for public needs in order to enhance legitimacy of the response and facilitate the development of shared identity between communities and authorities, subsequently promoting increased adherence to recommended behaviors, for example mass testing [15,16].

Identity also plays a role in the sense of responsibility duty that was frequently cited by Liverpool residents for reasons why they were engaging in testing. This response is not unique to Liverpool residents; in a recent survey of university students taking part in asymptomatic testing, the majority of students stated they took part in testing because they wanted to protect others (91%) and because it was the right thing to do (82%). A smaller proportion (63%) also stated they took part to help fight the virus [17]. Return to normality was also identified as a key motivator to engage in testing. This has also been identified elsewhere; for example, in the pilot in Slovakia, a relaxation of restrictions was offered as an incentive to participate and increased willingness to take part in the pilot.

However, it is not enough that people are willing to take part in mass testing; they must also be able to do so. Our analysis of local narratives in Liverpool identified several structural barriers, which made it more challenging to access testing, even for those willing to engage in the pilot. These were primarily access to testing sites and queues, for example not wanting to spend time traveling to a test site or waiting in a queue. Clear guidance about how to access testing and test sites would help negate concerns over access, for example dedicated websites or booking systems for asymptomatic testing where applicable; maps of where testing sites are located, including directions for how to access them (for example bus routes, nearest available public car park); and clear signage at the site.

In addition to being identified as an access barrier, queues were also cited as a barrier due to concern over risk of transmission. This was particularly early on in the pilot, where there was confusion between how to access asymptomatic testing opposed to the symptomatic testing to which the community had become accustomed. Requesting people to queue in proximity to others is contra to the basic public health guidance on protective behaviors that has become the pervasive narrative throughout the pandemic response: social distancing. To address concerns about being unable to social distance while waiting for testing, communicating what measures have been put in place to ensure safe queuing is an essential part of the communications for asymptomatic testing.

Financial concerns around the requirement to self-isolate if a test was positive were also highlighted as a barrier to testing. Several people stated that they would be reluctant to take a test because they would not receive any financial support and would therefore struggle to self-isolate if they received a positive result. It is essential that everyone required to self-isolate has the financial support to do so without encountering financial hardship, in order to improve adherence both to self-isolation [18,19] and to related behaviors (eg, testing) and to mitigate against adverse effects on mental health [20]. It is essential that people are aware of support available to them if they are self-isolating (eg, financial support scheme for people required to self-isolate), as this will remove some of the financial barriers associated with undergoing mass testing.

It is currently unclear the extent to which mass asymptomatic testing had an impact on cases or hospitalizations in Liverpool. As of December 9, 2020, one-quarter of the city’s residents engaged in the pilot and took a lateral flow test. During this time, nearly 900 people were identified as positive [4]. Interestingly, the uptake of testing in Liverpool was considerably lower than a similar pilot in Slovakia, where nearly all eligible people engaged in testing [2]. This highlights the importance of evaluating acceptance of asymptomatic testing, specifically identifying barriers and motivators to undergoing mass testing. The work presented here could therefore provide valuable insights into barriers and facilitators to mass testing that could be used to inform the way in which these processes are managed in future and could potentially increase uptake with mass testing programs.

### Recommendations

Based on the findings presented here, we suggest that, in order to promote good uptake of mass testing, authorities should communicate openly and honestly with communities, particularly about the nature and purpose of mass testing; provide clear instructions around practical aspects of testing (eg, details of site locations, how to access testing); provide financial support for self-isolation; listen to and address public concerns; engage with communities in order to understand their experiences; and ensure that communities know that their views are being taken into account (eg, where community engagement is taking place and being used to inform the response, this should be communicated).

### Limitations

Although analysis of social media data and other online media can facilitate access to the perspectives of those who do not necessarily choose to participate in other types of research, there is the potential that the demographic composition of digital media users may differ from that of the wider population [21]. The first limitation of this study is therefore that we only collected the perspectives of people who opted to publish their thoughts online; consequently, the sample may not be representative of the wider population.

The pragmatic thematic analysis of a targeted sample of social and online media sites presented in this paper was carried out to provide rapid insights into public perceptions of mass testing. A second limitation of the study is therefore that the rapid nature of the research meant that there was no time to carry out checks of interrater reliability. We recommend that future studies employ web scraping tools to capture a greater quantity of data and that checks of interrater reliability are carried out wherever possible. While every effort was taken to increase the likelihood that comments collected in the data set were all expressed by Liverpool residents, there is no guarantee that the data set was entirely limited to Liverpool residents.

### Conclusion

This study has highlighted several key barriers and facilitators to engaging in asymptomatic testing in residents in Liverpool city, including concerns over access, risk of transmission, and financial hardship. These structural barriers are amenable to mitigation and should be considered when rolling out similar testing programs elsewhere. We also identified psychosocial barriers, including lack of trust in authorities, which was associated with a sense of marginalization and disengagement with the testing program. This emphasizes the importance of recognizing and engaging with local community identity when implementing asymptomatic testing programs. We suggest that future implementation of mass testing programs should include honest and open communication to encourage and harness existing community identities, thereby enhancing the legitimacy of asymptomatic testing as a policy. In addition, adequate and accessible financial support needs to be in place prior to the implementation of community asymptomatic testing to mitigate any concerns surrounding financial hardship. Rapid thematic analysis of digital media is a pragmatic method to gather insights from communities around acceptability of public health interventions, such as mass testing or vaccination uptake. This methodological approach can complement other, more established approaches to ascertaining insights, such as surveys, interviews, and focus group discussions.

## Abbreviations

LCC: Liverpool City Council
MAST: mass, asymptomatic, serial testing
NHS: National Health Service
NIHR HPRU: National Institute for Health Research Health Protection Research Unit
PHE: Public Health England
UKHSA: United Kingdom Health Security Agency
